# Chemical and Physical Implications of the Use of Alternative Vessels to Oak Barrels during the Production of White Wines

**DOI:** 10.3390/molecules26030554

**Published:** 2021-01-21

**Authors:** Mariona Gil i Cortiella, Cristina Ubeda, José Ignacio Covarrubias, V. Felipe Laurie, Álvaro Peña-Neira

**Affiliations:** 1 Instituto de Ciencias Químicas Aplicadas, Inorganic Chemistry and Molecular Material Center, Facultad de Ingeniería, Universidad Autónoma de Chile, Av. El Llano Subercaseaux 2801, San Miguel, Santiago 8910060, Chile; 2 Instituto de Ciencias Biomédicas, Facultad de Ciencias, Universidad Autónoma de Chile, el Llano Subercaseaux 2801, San Miguel, Santiago 8910060, Chile; c_ubeda@us.es; 3 Área de Nutrición y Bromatología, Dpto. de Nutrición y Bromatología, Toxicología y Medicina Legal, Facultad de Farmacia, Universidad de Sevilla, C/P. García González n°2, E-41012 Sevilla, Spain; 4 Department of Agro-Industry and Enology, Facultad de Ciencias Agronómicas, Universidad de Chile, Santa Rosa 11315, La Pintana, Santiago 8820000, Chile; jcovarru@uchile.cl (J.I.C.); apena@uchile.cl (Á.P.-N.); 5 Facultad de Ciencias Agrarias, Universidad de Talca, 2 Norte 685, Talca 3465548, Chile; flaurie@utalca.cl

**Keywords:** concrete vessels, polyethylene vessels, clay jars, stainless-steel tanks, oval-shaped tanks, Sauvignon Blanc, white wine, aging on lees

## Abstract

Recently, the use of alternative vessels to oak barrels during winemaking has become increasingly popular, but little is known about their impact on the chemical composition of the resulting wines. To address this issue, a Sauvignon Blanc wine was elaborated from the same grape juice by using cylindrical stainless-steel tanks, oval-shaped concrete vessels, oval-shaped polyethylene vessels, and clay jars in triplicate. Each vessel was used for alcoholic fermentation and the aging of wines over its own lees. Wines elaborated in concrete vessels showed the highest pH and the lowest titratable acidity, most likely related to the observed release of inorganic compounds from the concrete walls. Little effect of the vessels was seen on the wine color and phenolic composition. Wines elaborated in clay jars showed the highest turbidity and the highest content of soluble polysaccharides, while those made using cylindrical stainless-steel tanks showed the highest content of volatile compounds. Despite the observed differences, all of the vessels tested seem suitable for white wine production since every wine showed chemical features that corresponded with the quality standards of Sauvignon Blanc wines.

## 1. Introduction

During the last decade, the use of different kinds of alternative vessels has become widespread in wineries. Such a trend includes fashionable oval vessels or classic jars made of inorganic materials, such as concrete or clay, or new polymeric substances such as technical polyethylene [1,2,3,4]. Several reasons justify the use of these vessels in wineries. First, they are used as an alternative to enhance geographical and varietal typicality, given that as opposed to oak-made vessels, they are not expected to release aroma compounds into wines [1,5]. Moreover, such vessels have relatively low capacities and allow winemakers to elaborate single-vineyard wines or premium wines by using small batches of high-quality grapes as the raw material [3].

As opposed to stainless steel tanks, concrete, clay, and polyethylene vessels are made of porous materials that allow oxygen to permeate through their walls [6], thus, thus possibly allowing the slow oxygenation of wines, somehow similar to that occurring in oak barrels [5]. Concrete vessels use to be coated with tartaric acid in order to develop a calcium tartrate (CaT) layer, which was later replaced by the use of chemically inert coatings such as epoxy resins [7]. However, due to the difficulties with the cleaning of vessels with CaT layers [8,9,10] or the release of potentially harmful compounds from epoxy resins [11,12] is that some winemakers are now using them free of coating, just like clay amphoras [13,14,15].

One of the main practices to elaborate premium white wines is aging on lees, usually performed in oak barrels. This practice basically consists of maintaining wines in contact with autolyzing yeasts for several months to modify the polysaccharide and lipid profiles of the resulting wines, thus improving some of their sensory attributes [16,17]. Several factors influence the impact of aging on lees including the following: contact time and temperature, yeast strain, turbidity of the fermentation medium, the resuspension of lees by stirring (oenological practice known as *batonnage*), or the use of rough racking of wines after alcoholic fermentation (to discard the coarse lees and perform wine aging with just fine lees) [17,18]. Currently, winemakers are combining the use of alternative vessels with the aging of white wine on lees, expecting the production of more distinctive wines.

Despite the increasing use of alternative vessels, scientific literature about the impact of such containers on the chemical composition of the resulting wines is still scarce [1,2,3,19]. To address this issue, a trial in which concrete oval-shaped vessels (OVO/CNCR), clay jars (JAR/CLAY), polyethylene oval-shaped vessels (OVO/PE), and conventional cylindrical stainless-steel tanks (CYL/INOX) were used in triplicate to ferment a Sauvignon Blanc wine, and the chemical, physical, and sensory characterization of the resulting stabilized wines was reported elsewhere [19], with no major differences among them. This lack of differences was supported by the limited period of time in which the wine remained in contact with the vessel materials and offered the basis to propose the current study. Given that wine aging on lees is a widespread oenological practice and that the use of vessel alternatives to oak barrels is increasing, we decided to assess whether the vessel used during aging on lees produces a meaningful impact on the final features of the resulting wines. To achieve this goal, wines fermented in the aforementioned vessels were maintained on their own lees for six months. After that, the wines were physically and chemically characterized to better understand the influence of the vessel used during winemaking on the resulting wines.

## 2. Results and Discussion

### 2.1. Impact of the Vessel on the Wine Acidity and Elemental Composition

As shown in Table 1, the wines fermented and aged in OVO/CNCR vessels showed the lowest titratable acidity and the highest pH among all treatments. However, no significant differences in the content of organic acids, potassium content, or conductivity were observed regardless of the type of vessel employed (Table 1). These seemingly diverging results may have to do with the higher contents of other cations observed in the OVO/CNCR wines, which may disrupt the equilibrium between tartaric acid and hydrogen tartrate forms in dissolution, leading to wines with a higher pH and lower titratable acidity (Table 1).

It should be noted that the wines were not subjected to a cold stabilization treatment, and all of them were unstable at the time of the analyses (according to the mini-contact test, a wine is considered unstable when the loss of conductivity is higher than 5%). However, wines elaborated in OVO/CNCR vessels showed the smallest conductivity loss, suggesting that the tartrate salts of OVO/CNCR wines were somewhat more stable when compared with the wines elaborated in the other vessels (Table 1). This result could be related to the greater concentration of magnesium observed for OVO/CNCR wines (with an increase of approximately 60–80% when compared with the other vessels) since it has been described that the presence of magnesium cations (Mg^2+^) increases the stability of tartrate salts [20,21,22].

The OVO/CNCR vessels used for this study were not coated with a chemically inert lining such as epoxy resins, thus the wines were in direct contact with the concrete material, which, as previously documented [23,24,25,26], may have released inorganic elements such as silicon, sodium, magnesium, iron, and manganese into the OVO/CNCR wines (Table 1). From these data, it seems that the enrichment of wines with these elements and the formation of inorganic salts could have altered the ionic strength and the equilibrium between tartaric acid and hydrogen tartrate forms in dissolution, leading to the changes observed in the pH and TA [25,27].

Considering that OVO/CNCR wines contain higher amounts of several of the elements studied in this trial, the lack of differences among the conductivities of the wines may be surprising (Table 1). However, as mentioned before, the wines were not cold stabilized, and as a consequence, they were supersaturated with potassium ions (K^+^), which could explain the lack of differences in conductivity [28]. A noteworthy increase in the content of iron in the wines elaborated in OVO/CNCR when compared with the wines made in other vessels (with an increase of about 400%) was observed. This concentration is far from posing a metal instability risk but could have contributed to flavor changes via catalytic oxidative reactions [29,30]. This type of iron release from non-coated concrete vessels has been previously described [26].

The release of inorganic compounds into the wines could also be expected for JAR/CLAY tanks [31]; however, among the analyzed elements, JAR/CLAY wines only show higher amounts of copper when compared with the other wines. This is in agreement with a study conducted in Qvevri wines, suggesting that processing on clay amphoras could result in a metal composition equivalent to that of conventional wines [14]. The copper concentration found does not imply a metal instability risk, but the function of copper as a catalyst for oxidative reactions may be a matter of concern [29]. In any case, there is a great diversity of mineralogical compositions for concrete and clay [32] and if a release of inorganic compounds from vessels to wine could take place, the specific chemical composition of the concrete or clay of which the vessels are made could impact the extent of the extraction phenomenon.

In addition, the results may also be influenced by the vessel shape and size as well as its surface-to-volume ratio, which determines the extent of the release of new elements into wine and the extent of their dissolution as inorganic salts. In this case, the vessel surface to wine volume ratios for OVO/CNCR and JAR/CLAY are quite similar: OVO/CNCR vessels have 79.1 cm^2^ per liter of wine (approximately 126 L of wine per m^2^ of concrete surface), and JAR/CLAY vessels have 82.0 cm^2^ per liter of wine (approximately 122 L of wine per m^2^ of clay surface). Thus, the differences in the release of inorganic compounds between the OVO/CNCR and JAR/CLAY vessels do not seem to be related to differences in the contact surface in this case.

### 2.2. Impact of the Vessel on the Wine Color and Phenolic Composition

As shown in Table 1, no significant differences were observed for the color parameters of wines, with the only exception of the red-green CIELab coordinate (a*), which was slightly greater for the OVO/PE and OVO/CNCR wines when compared with JAR/CLAY wines. In fact, differences in color among the wines are almost nonexistent and could not be observed by the naked eye [33]. The lack of differences in the color intensity and lightness (L*) among vessels may indicate similar rates of wine oxidation, in line with the expected increase in absorbance at 420 nm observed during the oxidation process of white wines [34]. Moreover, regarding the total phenols by a spectrophotometric approach (I_280_), JAR/CLAY wines showed the highest total phenolic content, followed by OVO/PE wines. However, when the phenolic composition was analyzed by RP-HPLC-DAD after SPE extraction from the wine matrix, only small differences were observed. Thus, the differences observed among the total phenolic contents are related to the method used, which corresponds to the optical density of diluted wine at 280 nm [35], and as a result of the low content of phenolic compounds, it is possible that proteins, glycoproteins, and inorganic compounds greatly contribute to wine absorbance at 280 nm [36].

Among all of the phenolic families described in white wines [37], only hydroxycinnamic acids were detected and quantified by SPE followed by RP-HPLC-DAD analysis, while neither flavonols nor stilbenes detected at quantifiable amounts. The quantification results of the hydroxycinnamic acids are shown in Table 2. According to these results, no differences in the total hydroxycinnamic acid content were found among wines elaborated in the different kinds of vessels. Slight differences could be observed regarding the esterification pattern of hydroxycinnamates. OVO/CNCR wines showed the highest ethyl ester content and the lowest tartaric acid ester content. Thus, regarding the color and phenolic composition of the wines, it seems that the vessels used during winemaking did not have a significant impact on the phenolic content. This is consistent with previous results in which stabilized wines were analyzed immediately after alcoholic fermentation [19].

### 2.3. Impact of the Vessel on the Wine Turbidity and Polysaccharide Content

Although not scientifically proven, one of the benefits attributed to oval-shaped vessels for wine production is that such a shape would favor the formation of convection currents inside the liquid, thus preventing suspended solids from settling at the bottom of the vessel and favoring the release of polymeric carbohydrate substances from suspended solids into the wine. Like so, it has been described that the shape of the bottom of the vessels influences the contact surface between wine and the settled solids [3]. The turbidity results shown in Table 1 agree with this idea, since the cylindrical vessels (CYL/INOX, flat-bottomed) showed the lowest turbidity of all, whilst the wines elaborated in JAR/CLAY, having an inverted oval shape, showed the highest turbidity results. Therefore, the results seem to agree with the hypothesis that oval-shaped vessels increase the suspended solids of wines regardless of the vertical orientation of the ovoid.

At this point, it should be noted that in this trial, aging on lees was performed without racking off the gross lees, mainly for two different reasons: On the one hand, winemakers are increasingly worried about the sustainability of wine production and the use of environmentally friendly practices in wineries. In that sense, racking the wines implies the use of greater amounts of water due to vessel cleanup requirements. On the other hand, it has been described that the use of aging on lees without racking enriched the mannoproteins of chardonnay wine while contributing to the solubilization of grape polysaccharides [17].

**Figure 1:**
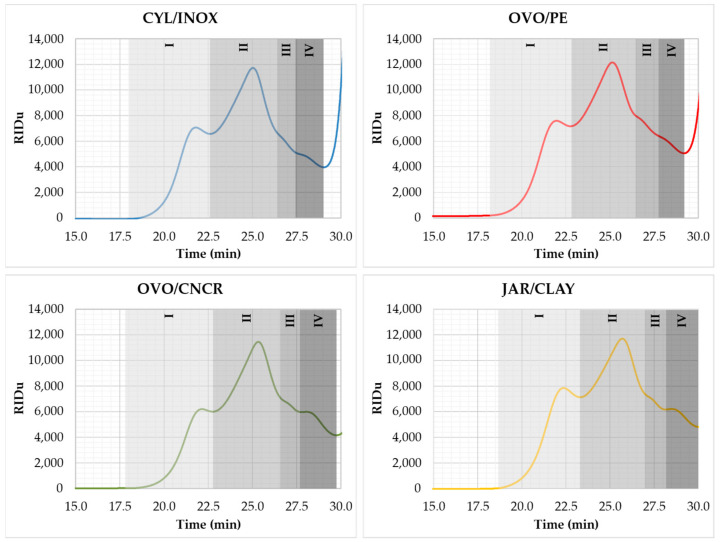
HRSEC-RID profile of wine soluble polysaccharides. The figure shows the averaged chromatograms of the triplicates for each kind of vessel. Gray quadrates indicate the range of each of the quantified fractions.

The soluble polysaccharides of wines were analyzed by High Resolution Size Exclusion Chromatography coupled to a Refraction Index Detector (HRSEC-RID) after their precipitation with cold acidified ethanol. Regarding the soluble polysaccharide profiles, some differences could be observed among the wines fermented and aged in the different kinds of vessels (Table 3, Figure 1). Briefly, the JAR/CLAY wines showed a lower average molecular mass (M_n_) for all of the studied fractions, while the CYL/INOX wines showed a higher average molecular mass (M_n_) for all of the studied fractions. OVO/PE and OVO/CNCR wines showed intermediate molecular weights, but statistical differences were mainly observed between CYL/INOX and JAR/CLAY wines. It has been previously reported that aging on lees resulted in the degradation of grape berry polysaccharides such as arabinogalactans and arabinans [18]. The results seem to indicate that the type of vessel used during wine aging could impact the rate of hydrolysis of wine polysaccharides, maybe due to a greater enzymatic activity from yeast lees.

The total polysaccharide content was greater for wines fermented and aged in clay jar vessels, mainly due to the higher content of FI (the polysaccharides with the greatest molecular mass) and FIV (the oligosaccharide fraction), given that no significant differences were observed among the wines for FII (the polysaccharides with a medium molecular mass) or FIII (the polysaccharides with low molecular mass). It has been previously reported that during aging on lees, wines were enriched with large polysaccharides (100–120 kDa) [38], and no differences on large polysaccharides were reported at the end of alcoholic fermentation [19]. Thus, the differences observed among the FI of wines fermented and aged in the different kinds of vessels were probably related to the aging on lees period. Although the wines fermented and aged in CYL/INOX vessels showed the lowest turbidity, they contained more large polysaccharides (FI) than OVO/CNCR wines; thus, the correlation between the suspended solids during aging and wine polysaccharide content does not seem as clear as winemakers expected. To clarify this issue, a statistical correlation analysis (using Pearson’s approach) among polysaccharide fractions and wine turbidity was performed (Table 4). The correlation between the wine turbidity and soluble polysaccharide content was very weak (*p* > 0.05) for the FI, FII, FIII, and total polysaccharide contents, while the correlation between the wine turbidity and FIV showed a Pearson’s correlation coefficient of 0.82 (*p* < 0.05). Thus, results suggest that higher turbidity during aging on lees could enhance the content of oligosaccharides in the resulting wines. A correlation between yeast macromolecule release during alcoholic fermentation and the turbidity of fermentation media has been previously described [39]. However, no differences in the wine turbidity or polysaccharide content were observed under the conditions of a previous study, in which the wines were analyzed immediately after alcoholic fermentation [19]. Therefore, the differences in turbidity and polysaccharide content observed in this study should be mainly attributed to the changes generated during aging over the lees period. Guilloux-Benatier and colleagues stated that the production of yeast macromolecules depends on the initial content of colloids prior to alcoholic fermentation, while they did not find evidence of higher enrichment with polysaccharides when the turbidity was higher during yeast autolysis [39].

### 2.4. Impact of the Vessel on the Volatile Composition of Wine

**Figure 2:**
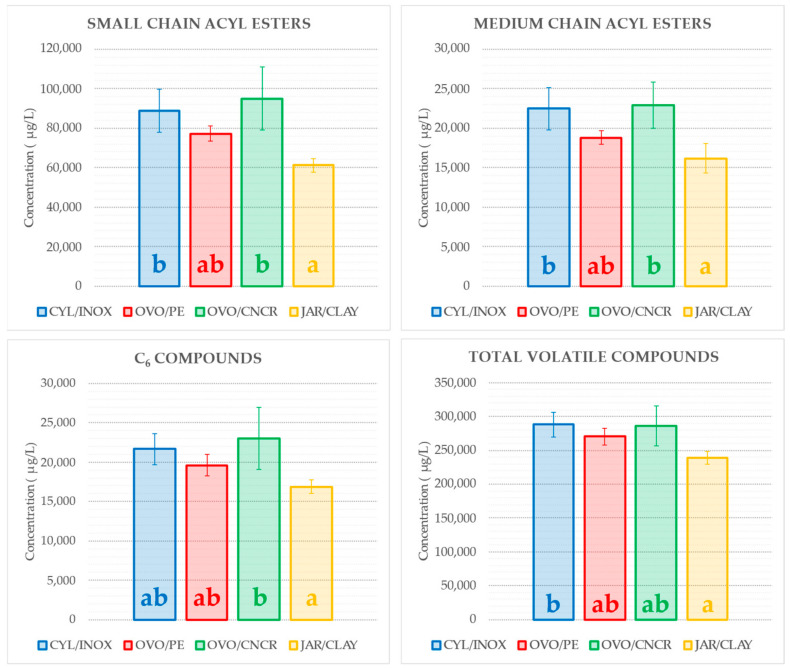
Contents of C_6_ compounds (including alcohols, acids, and esters), small chain acyl esters (including esters containing chains with 3 to 5 carbons), medium-chain acyl esters (including esters containing chains with 6 to 12 carbons), and total volatile compounds for each kind of vessel. Bars correspond to the average of the three replicates, and error bars correspond to their standard deviation. Different letters among bars indicate significant differences (*p* < 0.05) among wines.

The wines fermented and aged in the different kinds of vessels were subjected to SPME-GC-MS analysis to determine their volatile composition profiles (Table 5). Forty-four volatile compounds were identified and quantified, including 23 esters, eight alcohols, three acids, one aldehyde, eight terpenes (six monoterpenes and two sesquiterpenes), and one nor-isoprenoid. In general terms, the wines fermented and aged in JAR/CLAY vessels showed lower contents of several of the quantified volatile compounds. Regarding the volatile compound contents grouped by chemical family, significant differences were only found for acetates, other esters (all esters other than ethyl esters and acetates), and carboxylic acids, while no differences were observed for ethyl esters, alcohols, or terpenes. Considering acetates, other esters, and carboxylic acids, the CYL/INOX and OVO/CNCR wines showed the highest contents, while the JAR/CLAY wines showed the lowest. In fact, JAR/CLAY wines showed a statistically lower content of total volatile compounds (Figure 2) than CYL/INOX wines.

Although a sensory analysis of samples was not performed, the higher content of volatile compounds could indicate a higher potential aromatic intensity for CYL/INOX wines. However, it is well-known that some of the volatile compounds with high impact on wine sensory perception were found in very small concentrations in the wine matrix, and the correlation between the analytical determination of volatile compounds and wine sensory perception is much more complex than could be expected [40].

From a qualitative and quantitative point of view, esters are the major family of volatile compounds released during autolysis, beginning after approximately four months of aging [40]. Usually, short-chain (C3–C5) and medium-chain (C6–C12) acyl esters increase at the beginning of this lytic process [41]. Given that the raw grapes and wine production were the same for all the vessels, the differences in short-chain and medium-chain esters observed among wines could be attributed to the kind of vessel used during winemaking (Figure 2).

Several compounds released from lees can interact with volatiles, modifying the perception of the wine aroma. Thus, the release of fatty acids from yeasts into wine triggers the synthesis reactions of esters and aldehydes [42], or the increase in enzymes with esterase activity in the media may even alter the volatile profile of wines, mainly affecting fruity nuances [43]. Moreover, yeast mannoproteins may interact with aroma compounds and decrease their volatility [44], which leads to a reduction in the volatile compound concentration when determined by GC-MS [45,46]. A strong binding capability of volatile compounds by the insoluble fraction of yeast autolysates has been described [45,47,48]. The reported results agree with this idea since the wines produced in JAR/CLAY wines showed the highest turbidity values (mostly due to insoluble yeast fractions) and lower contents of volatile compounds (Table 5, Figure 2). Moreover, a negative correlation between the ester fractions (both small chain and medium-chain esters) and the polysaccharide contents is observed, also suggesting the loss of aroma compounds due to the presence of insoluble solids (Table 4). These data suggest that an adsorption phenomenon of volatile compounds by yeast (and/or grape) cell walls took place during winemaking.

**Figure 3:**
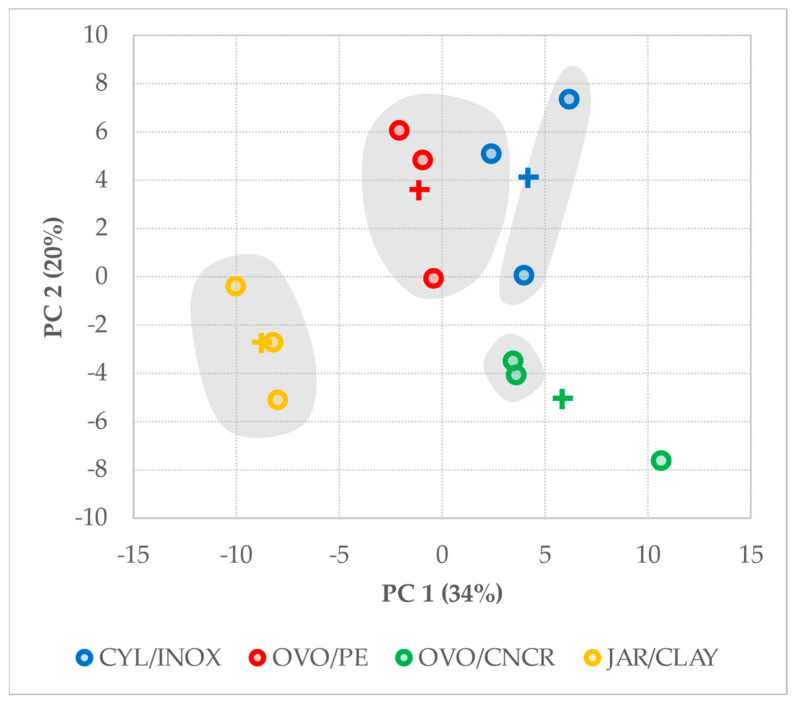
Score values resulting from a principal component analysis of all the data collected on wine samples. + indicates the centroid for each triplicate. Gray globes grouped wines according to a cluster analysis.

Despite the difficulty of predicting wine sensory properties from its volatile compound profile, C6 compounds have been largely related to vegetal scents [49]. As shown in Figure 3, the JAR/CLAY wines showed the lowest amount of C6 compounds, while the OVO/CNCR wines showed the greatest. In addition, JAR/CLAY wines also showed the highest amount of ethyl heptanoate (Table 5), which corresponds to a fruity ester compound with an odd carbon backbone that comes from grape precursors since they cannot be synthesized by yeasts during alcoholic fermentation [50]. Regarding such differences in volatile profiles of wines from the different tanks, it seems that the use of different vessels could be a useful tool to modulate the wine aromatic profile, enhancing or masking its fruity or vegetal scents.

### 2.5. Multivariate Analysis

Given the high amount of collected chemical data, a multivariate statistical analysis was performed (principal component analysis, PCA) to understand whether variability among wines is explained by the kind of vessel used during alcoholic fermentation and aging over lees. As Figure 3 shows, only 54% of the variability observed is explained by the two main principal components (PC 1 explains 34% of the variance and PC 2 explains the remaining 20%), but 116 variables were used to perform the multivariate analysis. Gray globes in Figure 3 show sample grouping according to Euclidean cluster analysis by using the same 116 variables. According to the multivariate analyses, JAR/CLAY wines differ from the others since they were cluster-grouped in the negative region of PC 1. The main reason for that aggregation (the loadings for each variable resulting from PCA can be found in Table 6) is their lower content of most of the analyzed volatile compounds in addition to their hydroxycinnamic acid and polysaccharide profiles. OVO/PE wines showed negative PC 1 scores, as happened with JAR/CLAY wines. However, OVO/PE wines are not distinguishable from CYL/INOX wines or OVO/CNCR wines by the PC 1 score distribution. Regarding PC 2, which explains 20% of the data variability, an interesting distribution of scores is observed when the vessel’s material is considered. JAR/CLAY and OVO/CNCR wines, corresponding to the noninert materials (they could be attacked by acidic solutions such as wine releasing some inorganic compounds consequently), showed negative PC 2 scores. In contrast, CYL/INOX and OVO/PE wines, corresponding to inert materials (they could not be attacked by acidic solutions such as wine), showed positive PC 2 scores. This distribution suggests that the material of which the vessel is made has an impact on the overall chemical composition of the resulting wine. Therefore, under the conditions of the trial, the fermenting and aging of lees wines in clay jars led to a more distinctive chemical composition than when the other kinds of vessels were used.

## 3. Materials and Methods

### 3.1. Experimental Design

Sauvignon Blanc grapes from the Leyda Valley (Chile) [33°54′4.568″ S; 71°44′0.841″ W], with approximately 12 t/ha fruit yield, were hand-harvested on April 16, 2018, and transported to the Miguel Torres Winery (Maule, Curicó, Chile). Grapes were destemmed, crushed, and pressed at approximately 65% juice yield. Then, the juice was subjected to a settling period of 24 h (12–14 °C) in a stainless-steel tank of 50,000 L, followed by racking into the different tanks used for this trial (i.e., cylindrical stainless-steel tanks, oval-shaped concrete vessels, oval-shaped polyethylene vessels, and clay jars). All juices were inoculated with 0.2 g/L of a mix of commercial starter yeasts (i.e., 50% ANCHOR VIN 13 and 50% ANCHOR VIN 7, from Oenobrands/Anchor Oenology, Montpellier, France). The Sauvignon Blanc juice employed had 22.1° Brix, 6.75 g/L (tartaric acid equivalents) titratable acidity, a pH of 3.4, and 174 mg/L yeast assimilable nitrogen (YAN). Four different commercially available tanks were used in triplicate (hence, a total of twelve tanks were used for this assay): [CYL/INOX], 150 L stainless steel tanks (Navarro y Cia. Ltd.a., Santiago, Chile); [OVO/PE], 980 L oval-shaped polyethylene tanks (Apollo FLEXTANK, Partner Ltd., Santiago, Chile); [OVO/CNCR], 450 L oval-shaped concrete tanks (De Navarra Ltd., Curicó, Chile); and [JAR/CLAY], 225 L clay jars (Value Juices Ltd., Santiago, Chile). Concrete vessels were treated with aqueous tartaric acid solutions following the instructions of the manufacturer previously to start the winemaking trials. The approach used to calculate the inner surface of OVO/CNCR and JAR/CLAY vessels consists of considering them as revolution ellipsoids. All tanks were placed in an underground cellar at a controlled temperature of 18 ± 1 °C, and the fermentation process was controlled daily by measuring the density and temperature of the musts. The alcoholic fermentation was considered complete when the density remained constant for at least two days and the residual sugar concentration was below 2 g/L. Once the alcoholic fermentation was finished, wines were sulfited (200 mg K_2_S_2_O_5_/L) and maintained in the same vessels for six months (18 ± 1 °C) of aging on their own lees. A single *batonnage* of resuspended yeast lees was performed at half of the aging time (three months after the end of alcoholic fermentation). After the six months of aging time had elapsed, a sample of 5 L from each wine was taken, subjected to a settling period of two days (18 ± 1 °C), bottled in green glass bottles (750 mL) and capped with crown caps. Bottles were stored in a dark cellar (16 ± 1 °C) until chemical and physical analyses that started two months after bottling.

### 3.2. General Analyses

General parameters of wines (i.e., alcohol content, titratable acidity and pH) were analyzed according to the analytical methods recommended by the International Organization of Vine and Wine [51]. A METTLER TOLEDO Seven Compact pH/ion meter (Mettler Toledo, Columbus, OH, USA) was used for pH measurements. The spectrophotometric measurements (i.e., color intensity, CIELab coordinates, and phenolic index (I_280_)) of the wines were analyzed as previously reported [19] by using a SHIMADZU UV-1900 UV-Vis spectrophotometer (Shimadzu Latin America S.A., Montevideo, Uruguay). Turbidity measurements were performed by using a HANNA HI 88731-ISO turbidimeter (HANNA Instruments Chile, Santiago, Chile), and conductivity measurements were performed by using a HANNA HI 5321 conductometer (HANNA Instruments Chile, Santiago, Chile). The mini-contact test [52] was used to check the tartrate potential instability of wines.

### 3.3. Organic Acids Analysis

Organic acids analysis was performed by using an Agilent 1260 Infinity Series chromatograph (Agilent Technologies, Santa Clara, CA, USA) equipped with a quaternary pump, an autosampler, a column oven, and a diode array detector following the method previously described [19]. The three main organic acids of wine (tartaric acid, malic acid, and citric acid) were identified and quantified by using their respective commercial standard (Sigma-Aldrich, Santiago, Chile).

### 3.4. Elemental Analysis

The wine composition for macro- (K, P, Mg, Ca, Si, Na and B) and microelements (Fe, Mn, Zn and Cu) was determined by MP-AES 4200 microwave plasma atomic emission spectroscopy (Agilent Technologies, Santa Clara, CA, USA) after acid digestion of the wine samples [19].

### 3.5. Individual Low-Molecular-Mass Phenolic Compound Determination

Low-molecular-mass phenolic compounds were extracted by solid-phase extraction (SPE) using Oasis^®^ MCX 6 cc (500 mg) cartridges (Waters, Iadet, Santiago, Chile) as reported [19], and analyzed by RP-HPLC-DAD by using the same equipment reported in Section 3.3 and a Zorbax Eclipse XDB-C18 Narrow-Bore (2.1 mm × 150 mm, 3.5 μm particle size. The identified and quantified compounds were: caftaric acid, coutaric acid, fertaric acid, *cis*-coumaric acid, *trans*-coumaric acid, caffeic acid, ferulic acid, sinapic acid, ethyl caffeoate, and ethyl coumarate. The identification of compounds was performed with commercial standards (when available). When commercial standards were not available, the retention time and UV-vis spectra were used. The quantification was performed by using caffeic acid as an external standard.

### 3.6. Volatile Compounds

The volatile compound profiles of wines were obtained by GC-MS after SPME as previously reported [19] using an Agilent 7890B gas chromatograph coupled to an Agilent 5977 Inert quadrupole mass spectrometer (Agilent Technologies, Santa Clara, CA, USA) equipped with a Gerstel MP2 autosampler (Gerstel, Mülheim an der Ruhr, Germany). A DB-WAX capillary column (60 mm × 0.25 mm, 0.25 μm film thickness; Agilent Technologies, Analitica Weisser, Santiago, Chile) was used with helium as the carrier gas at a flow rate of 1 mL/min. The identification of the compounds was based on comparison with authentic reference standards. When the standard was unavailable, tentative identification was carried out by comparing the mass spectra obtained from each molecule with the reference spectra of the NIST 98 software library and comparing their retention index (LRI) values with the literature. Data are expressed as concentrations (μg/L) obtained from calibration curves with reference standards. In the cases of unavailable standards, they were quantified with compounds of the same chemical family with the same major ion.

### 3.7. Soluble Polysaccharide Content of Wines

Polysaccharides were estimated by means of HRSEC-RID after precipitation with cold acidified ethanol [19].

### 3.8. Statistical Analysis

All results are expressed as the arithmetic mean ± standard deviation of three replicates. One-way analysis of variance (ANOVA) (*p* < 0.05) was performed with Infostat software (v. 2018), and the Student–Newman–Keuls post-hoc test was used for multiple comparisons and computed with the same software for physical and chemical data. Pearson’s correlation analysis among polysaccharide fractions and turbidity was performed with the same software as well as multivariate statistics: principal component analysis and cluster analysis (average linkage by the Euclidean approach).

## 4. Conclusions

In view of the prior results, it should be noted that the different kinds of vessels used to perform this trial were suitable for the production of chemically sound Sauvignon Blanc. According to the experimental conditions used, winemaking using clay jars seems to favor the development of more chemically distinctive wines than winemaking using other kinds of vessels. Given the results of a prior experiment, in which few differences among wines were observed, we could also suggest that longer periods of storage inside the vessels are required to observe significant changes in the chemical features of the wines [19], especially in regard to the wine turbidity and polysaccharide contents. However, it should be noted that the reported differences among wines made in the different vessels are very low if the overall chemical variability of white wines is considered. The magnitude of the differences reported suggests that the use of different kinds of vessels could help winemakers modulate some final features of wine, but the extent of such intervention is limited since the main features of the resulting wines depend much more on the raw grapes and winemaking practices than the type of vessel utilized.

## Figures and Tables

**Table 1:** General parameters and elemental composition of wines processed in different types of vessels, after six months of aging on their own lees.

Parameter ^(1)^	CYL/INOX	OVO/PE	OVO/CNCR	JAR/CLAY
Ethanol (% vol.)	13.4 ± 0.1	13.3 ± 0.1	13.4 ± 0.2	13.3 ± 0.1
pH	3.32 ± 0.01 a	3.31 ± 0.01 a	3.38 ± 0.01 b	3.28 ± 0.01 a
Titratable acidity ^(2)^	7.06 ± 0.25 b	7.20 ± 0.04 b	6.42 ± 0.21 a	6.90 ± 0.06 b
Tartaric acid (g/L)	3.40 ± 0.16	3.49 ± 0.05	3.49 ± 0.41	3.50 ± 0.03
Malic acid (g/L)	3.27 ± 0.11	3.40 ± 0.01	3.29 ± 0.31	3.49 ± 0.05
Citric acid (g/L)	0.68 ± 0.14	0.79 ± 0.01	0.70 ± 0.04	0.72 ± 0.01
Conductivity loss (%) ^(3)^	7.6 ± 0.7	7.6 ± 1.0	6.6 ± 0.6	7.3 ± 1.1
Conductivity (µS cm^−1^)	1.77 ± 0.03	1.81 ± 0.05	1.81 ± 0.05	1.80 ± 0.03
Turbidity (NTUs)	3.88 ± 0.87 a	5.58 ± 0.18 b	6.35 ± 0.90 bc	7.48 ± 0.76 c
Potassium (K, mg/L)	605 ± 30	598 ± 21	607 ± 19	582 ± 36
Phosphorous (P, mg/L)	115 ± 2 b	124 ± 3 c	112 ± 2 b	106 ± 5 a
Calcium (Ca, mg/L)	61 ± 3 b	63 ± 2 b	38 ± 5 a	57 ± 7 b
Silicon (Si, mg/L)	32 ± 7 a	27 ± 1 a	61 ± 11 b	26 ± 1 a
Sodium (Na, mg/L)	14.9 ± 2.0 a	17.1 ± 0.8 a	22.4 ± 4.5 b	16.1 ± 0.5 a
Magnesium (Mg, mg/L)	10.8 ± 1.9 a	8.9 ± 4.0 a	60.6 ± 27.5 b	19.3 ± 9.5 a
Boron (B, mg/L)	5.24 ± 0.29	5.07 ± 0.37	4.59 ± 0.38	4.56 ± 0.20
Iron (Fe, mg/L)	0.520 ± 0.061 a	0.433 ± 0.021 a	2.447 ± 0.341 b	0.577 ± 0.110 a
Manganese (Mn, mg/L)	0.623 ± 0.021 a	0.627 ± 0.006 a	0.737 ± 0.049 b	0.617 ± 0.032 a
Zinc (Zn, mg/L)	0.590 ± 0.010	0.550 ± 0.020	0.690 ± 0.150	0.767 ± 0.106
Copper (Cu, mg/L)	0.107 ± 0.012 a	0.110 ± 0.010 a	0.093 ± 0.015 a	0.170 ± 0.040 b
I_280_^(4)^	9.03 ± 0.15 a	9.57 ± 0.14 b	9.06 ± 0.19 a	9.99 ± 0.30 c
Color Intensity	0.124 ± 0.020	0.124 ± 0.011	0.144 ± 0.014	0.142 ± 0.009
L* ^(5)^	98.7 ± 0.3	98.6 ± 0.2	98.2 ± 0.2	98.3 ± 0.1
a* ^(6)^	−1.52 ± 0.06 ab	−1.45 ± 0.06 b	−1.47 ± 0.01 b	−1.62 ± 0.08 a
b* ^(7)^	8.11 ± 1.17	7.80 ± 0.62	8.61 ± 0.94	8.59 ± 0.69

^(1)^: Different letters in a row indicate significant differences (*p* < 0.05) among wines. ^(2)^: Titratable acidity expressed as tartaric acid equivalents (g/L). ^(3)^: Conductivity loss (expressed as a percentage) resulting from applying the mini-contact test. ^(4)^: Index of the total phenolic content. ^(5)^: Lightness coordinate of CIELab space. ^(6)^: Red-green axis coordinate of CIELab space. ^(7)^: Yellow-blue axis coordinate of CIELab space.

**Table 2:** Hydroxycinnamic acid analysis after six months of aging of wines on their own lees.

Structure ^(1)^	CYL/INOX	OVO/PE	OVO/CNCR	JAR/CLAY
Caffeic acid and derivatives	145.1 ± 4.2	143.3 ± 2.98	144.3 ± 2.4	151.2 ± 2.2
Coumaric acid and derivatives	74.1 ± 4.3	79.0 ± 2.67	72.8 ± 2.2	75.0 ± 2.7
Ferulic acid and derivatives	4.30 ± 0.49	3.85 ± 0.44	4.50 ± 0.88	4.19 ± 0.62
Sinapic acid and derivatives	2.26 ± 0.26	2.53 ± 0.16	2.53 ± 0.21	2.34 ± 0.03
Free cinnamic acids	54.5 ± 2.5	57.1 ± 0.5	57.5 ± 1.0	54.8 ± 0.7
Tartaric esters of cinnamic acids	159.6 ± 4.1 ab	157.2 ± 4.7 ab	150.7 ± 2.9 a	164.0 ± 1.0 b
Ethyl esters of cinnamic acids	14.3 ± 0.9 a	14.4 ± 0.2 a	16.0 ± 0.7 b	13.9 ± 0.6 a
Total cinnamic acids and derivatives	225.8 ± 6.9	228.7 ± 5.0	224.2 ± 1.8	232.7 ± 1.6

^(1)^: Different letters in a row indicate significant differences (*p* < 0.05) among wines. The results are expressed as mg/L caffeic acid equivalents. The results for each cinnamic acid include the free form of the acid and their respective esters.

**Table 3:** Polysaccharide soluble fractions determined by HRSEC-RID after precipitation with cold acidified ethanol of wines after six months of aging on their own lees.

FRACTION	Parameter ^(1)^	CYL/INOX	OVO/PE	OVO/CNCR	JAR CLY
I	mg/L	64.94 ± 6.59 b	70.53 ± 3.84 bc	52.61 ± 4.05 a	79.28 ± 3.54 c
	Range (KDa)	600.42–60.33	577.82–55.84	694.23–56.12	515.64–42.46
	M_n_ (KDa) ^(2)^	92.22 ± 6.53 b	86.49 ± 8.92 b	77.81 ± 4.42 ab	69.40 ± 5.33 a
II	mg/L	148.67 ± 7.78 a	155.67 ± 3.99 a	146.71 ± 2.23 a	154.13 ± 5.37 a
	Range (KDa)	60.33–9.69	55.84–8.95	56.12–8.39	42.46–6.63
	M_n_ (KDa) ^(2)^	17.65 ± 1.22 b	16.51 ± 2.06 b	15.13 ± 1.09 ab	12.64 ± 1.17 a
III	mg/L	32.91 ± 6.82 a	36.78 ± 2.12 a	34.97 ± 6.19 a	33.01 ± 2.78 a
	Range (KDa)	9.69–5.25	8.95–5.02	8.39–4.55	6.63–3.78
	M_n_ (KDa) ^(2)^	9.69 ± 0.60 b	8.53 ± 1.47 ab	8.01± 1.54 ab	6.63 ± 0.50 a
IV	mg/L	33.52 ± 4.20 a	43.42 ± 3.99 b	47.33 ± 4.22 b	50.12 ± 3.53 b
	Range (KDa)	5.25–2.29	5.02–2.10	4.55–1.61	3.78–1.38
	M_n_ (KDa) ^(2)^	5.25 ± 0.44 b	5.01 ± 0.73 b	3.76 ± 0.27 a	3.24 ± 0.44 a
**Total polysaccharides (mg/L)**		280.05 ± 18.80 a	306.41 ± 7.88 ab	281.62 ± 9.20 a	316.55 ± 11.41 b

^(1)^: Different letters in a row indicate significant differences (*p* < 0.05) among wines. ^(2)^: Averaged number molecular mass of each fraction.

**Table 4:** Pearson’s correlation analysis among polysaccharide fractions, ester fractions, and wine turbidity.

	F I	F II	F III	F IV	Total Polysaccharides	Small Chain Esters	Medium Chain Esters
**F II**	**0.6481** *	-					
**F III**	−0.0072	0.4465	-				
**F IV**	0.0834	0.2698	0.1974	-			
**Total polysaccharides**	**0.7790** **	**0.8652** ***	0.4355	0.5500	-		
**Small chain esters**	**−0.8147** **	**−0.6745** *	−0.3052	−0.3441	**−0.8546** ***	-	
**Medium chain esters**	**−0.8166** **	**−0.6170** *	−0.1536	−0.2987	**−0.7862** **	**0.9300** ***	-
**Turbidity**	0.2614	0.2795	−0.0561	**0.8216** **	0.5259	−0.3853	−0.3760

Numbers below the diagonal correspond to Pearson’s coefficient values. *p*-values were indicated as follows: * *p* < 0.05, ** *p* < 0.01, *** *p* < 0.001.

**Table 5:** Volatile compounds of wines expressed as μg/L.

Volatile Compound	LRI ^(a)^	ID ^(b)^	CYL/INOX	OVO/PE	OVO/CNCR	JAR/CLAY
Ethyl butyrate	1055	A	808.03 ± 96.81 b	699.60 ± 52.31 ab	832.61 ± 96.15 b	556.19 ± 110.41 a
Ethyl 2-methylbutyrate	1084	A	22.40 ± 2.98	21.74 ± 0.64	19.19 ± 1.03	20.59 ± 5.18
Ethyl 2-ethylbutyrate	1095	C	4.60 ± 0.35	5.01 ± 0.35	5.06 ± 1.42	3.35 ± 0.46
Isoamyl acetate	1122	A	85,474 ± 10,851 b	74,822 ± 3799 ab	91,334 ± 16,115 b	59,224 ± 3638 a
Ethyl hexanoate	1245	A	10,750 ± 1555	9707 ± 446	10,669 ± 2055	8912 ± 1261
Hexyl acetate	1285	A	5394.54 ± 763.22 b	4597.29 ± 106.79 ab	5566.26 ± 1322.53 b	3382.21 ± 400.09 a
*trans*-4-Hexenyl acetate	1295	C	52.80 ± 2.26 b	42.86 ± 4.39 b	57.56 ± 14.02 b	20.51 ± 5.77 a
*cis*-3-Hexenyl acetate	1320	C	196.48 ± 28.14	176.40 ± 12.31	201.47 ± 49.47	135.47 ± 17.64
Ethyl 2-hexenoate	1339	B	3.89 ± 0.18	3.45 ± 0.14	3.99 ± 0.56	3.28 ± 0.38
Ethyl heptanoate	1334	A	1.98 ± 0.08 a	1.82 ± 0.13 a	1.74 ± 0.26 a	2.87 ± 0.44 b
Ethyl octanoate	1437	A	2261.88 ± 393.31	1931.85 ± 233.56	2276.79 ± 308.22	1697.13 ± 277.35
Isoamyl hexanoate	1495	C	0.86 ± 0.44	0.75 ± 0.12	1.14 ± 0.41	0.68 ± 0.41
Methyl octanoate	1465	B	18.97 ± 2.29	17.81 ± 4.00	26.86 ± 2.97	16.84 ± 5.376
Ethyl nonanoate	1541	B	4.46 ± 1.06	3.32 ± 0.94	3.57 ± 0.99	4.02 ± 0.68
Methyl decanoate	1600	A	7.17 ± 0.79	5.98 ± 1.41	9.12 ± 2.03	5.94 ± 0.81
Ethyl decanoate	1647	A	810.80 ± 113.50 b	457.27 ± 40.36 a	705.49 ± 166.62 b	341.61 ± 62.49 a
Diethyl succinate	1675	A	40.39 ± 0.58	36.79 ± 8.81	30.97 ± 5.62	33.27 ± 3.44
Isoamyl octanoate	1680	A	1384.67 ± 248.97	1033.59 ± 89.61	1456.29 ± 95.94	985.56 ± 249.45
β-Phenylethyl acetate	1851	A	4947.58 ± 703.78	3844.20 ± 1042.93	4164.25 ± 1036.14	2823.48 ± 571.46
Ethyl dodecanoate	1864	B	536.12 ± 89.61	314.78 ± 32.71	624.74 ± 257.51	326.99 ± 23.53
Methyl dodecanoate	1825	B	2.83 ± 0.31 b	1.79 ± 0.16 a	2.25 ± 0.17 a	1.98 ± 0.45 a
Isoamyl decanoate	1888	A	1041.45 ± 182.94 b	508.70 ± 149.70 a	1329.18 ± 254.95 b	329.49 ± 94.03 a
Ethyl tetradecanoate	2041	B	20.85 ± 6.27 ab	20.05 ± 5.23 ab	28.79 ± 5.88 b	13.71 ± 1.48 a
Isobutanol	1074	A	32,457 ± 1164	33,883 ± 2188	27,862 ± 6915	31,451 ± 1515
Isoamyl alcohol	1200	A	110,845 ± 6122	111,657 ± 7618	113,832 ± 16572	109,864 ± 5907
Hexanol	1375	A	1275.30 ± 120.00	1050.00 ± 135.59	1084.08 ± 145.98	990.41 ± 40.46
2,3-Butanediol	1557	A	68.30 ± 4.37	67.32 ± 24.83	75.66 ± 41.31	54.08 ± 35.74
Decanol	1773	A	3.69 ± 0.31 b	3.02 ± 0.67 b	3.36 ± 0.65 b	1.43 ± 0.47 a
2-phenylethanol	1940	A	17664 ± 2227	15665 ± 3686	10779 ± 3165	10931 ± 5288
Dodecanol	1986	A	2.04 ± 0.26 b	1.03 ± 0.21 a	0.96 ± 0.07 a	1.15 ± 0.22 a
Benzaldehyde	1565	A	28.18 ± 13.27	7.98 ± 3.15	32.31 ± 12.83	16.80 ± 5.00
Hexanoic acid	1880	A	3710.25 ± 657.96	3780.41 ± 1061.81	5162.74 ± 836.42	3211.51 ± 955.41
Octanoic acid	2076	A	5808.19 ± 468.00 b	5448.26 ± 596.99 b	5852.82 ± 872.22 b	2800.26 ± 785.91 a
Decanoic acid	2339	A	2141.84 ± 303.72 b	628.51 ± 243.61 a	1793.36 ± 383.54 b	500.48 ± 51.18 a
TDN ^(c)^	1745	B	8.22 ± 1.02	6.69 ± 0.84	7.54 ± 2.18	6.06 ± 1.75
Nerol oxide	1462	B	44.05 ± 3.19	43.52 ± 4.98	41.57 ± 1.26	41.57 ± 7.49
Geranyl ethyl ether	1481	C	7.98 ± 1.40	8.01 ± 0.66	6.78 ± 0.32	6.49 ± 0.90
Linalool formate	1539	C	5.28 ± 0.61 b	4.66 ± 0.26 ab	4.80 ± 0.43 ab	3.77 ± 0.60 a
Linalool	1555	A	5.60 ± 1.08	5.61 ± 1.92	6.10 ± 1.53	5.90 ± 0.93
Hotrienol	1660	B	13.02 ± 0.61	12.68 ± 2.90	14.65 ± 1.53	14.94 ± 0.71
*cis*-β-Farnesene	1664	B	2.80 ± 0.31 b	2.37 ± 0.11 b	2.63 ± 0.32 b	1.67 ± 0.06 a
α-Terpineol	1693	A	7.35 ± 1.89	7.66 ± 0.93	7.27 ± 1.00	7.11 ± 1.05
*trans*-Nerolidol	2047	A	8.10 ± 0.86 b	6.81 ± 1.70 b	7.70 ± 1.34 b	3.41 ± 0.51 a
TOTAL ETHYL ESTERS			15,266 ± 2067	13,203 ± 801	15,203 ± 2025	11,915 ± 1649
TOTAL ACETATE ESTERS			96,065 ± 11,614 b	83,483 ± 2771 ab	101,323 ± 16,194 b	65,585 ± 4002 a
TOTAL OTHER ESTERS			2456 ± 149 b	1569 ± 75 a	2825 ± 287 b	1340 ± 218 a
TOTAL ALCOHOLS			162,588 ± 5896	162,553 ± 9513	153,910 ± 10,703	153,530 ± 11,813
TOTAL TERPENES			86.08 ± 4.89	84.52 ± 8.34	83.81 ± 2.70	81.47 ± 10.20
TOTAL ACIDS			11,660 ± 1016 b	9857 ± 1344 b	12,809 ± 1838 b	6512 ± 1530 a

Different letters in a row indicate significant differences (*p* < 0.05) among wines. ^(a)^ ID: reliability of identification: A, mass spectrum and LRI agreed with standards; B, mass spectrum agreed with the mass spectral database and LRI agreed with the literature data (Pherobase: www.pherobase.com; NIST Mass Spectrometry Data Center: https://webbook.nist.gov/); C, tentatively identified, mass spectrum agreed with the mass spectral database. ^(b)^ LRI: linear retention index. ^(c)^ Results (average ± SD) are expressed as µg/L except TDN results, which are expressed in relative area.

**Table 6:** Loadings were obtained for each variable contributing to the two main principal components extracted.

Variable	PC 1	PC 2
*trans*-4-Hexenyl acetate	**0.156**	−0.006
TOTAL ESTERS	**0.154**	−0.004
TOTAL ACETATE ESTERS	**0.153**	0.000
Isoamyl acetate	**0.151**	−0.009
Ethyl butyrate	**0.147**	−0.005
TOTAL ALIPHATIC C6 COMPOUNDS	**0.145**	−0.020
TOTAL VOLATILE COMPOUNDS	**0.143**	0.021
Hexyl acetate	**0.142**	0.013
TOTAL ACIDS	**0.140**	0.005
TOTAL ETHYL ESTERS	**0.136**	−0.022
TOTAL ALIPHATIC C8 ESTERS	**0.135**	−0.041
Octanoic acid	**0.135**	0.034
Ethyl 2-hexenoate	**0.135**	−0.039
Decanol	**0.135**	0.042
Farnesene	**0.134**	0.035
Ethyl octanoate	**0.133**	−0.030
TOTAL OTHER ESTERS	**0.129**	−0.043
*cis*-3-Hexenyl acetate	**0.126**	0.026
Decanoic acid	**0.125**	0.023
M_0_ FII	**0.125**	**0.102**
M_f_ FI	**0.125**	**0.102**
Ethyl 2-ethylbutyrate	**0.124**	−0.004
Isoamyl decanoate	**0.123**	−0.033
Ethyl decanoate	**0.120**	0.033
Ethyl hexanoate	**0.119**	−0.025
*trans*-Nerolidol	**0.117**	0.036
pH	**0.117**	**−0.100**
Linalool formate	**0.116**	0.059
Isoamyl octanoate	**0.109**	−0.048
Si	**0.107**	**−0.109**
TDN	**0.107**	−0.027
M_0_ FI	**0.103**	−0.030
Ethyl esters of cinnamic acids	**0.100**	**−0.129**
Isoamyl hexanoate	0.099	−0.056
M_0_ FIII	0.098	**0.128**
M_f_ FII	0.098	**0.128**
*cis*-3-Hexen-1-ol	0.095	0.002
M_n_ FII	0.095	**0.143**
M_0_ FIV	0.094	**0.132**
M_f_ FIII	0.094	**0.132**
Ethyl caffeoate	0.093	**−0.148**
Hexanol	0.093	0.087
a*	0.091	0.043
Mn	0.090	**−0.113**
Methyl octanoate	0.089	**−0.114**
Benzaldehyde	0.088	**−0.100**
Hexanoic acid	0.088	−0.050
Ethyl tetradecanoate	0.088	−0.053
M_n_ FIII	0.087	**0.114**
Fe	0.084	**−0.134**
Ethyl coumarate	0.080	−0.030
M_n_ FI	0.080	**0.159**
β-Phenylethyl acetate	0.078	**0.120**
Methyl decanoate	0.077	−0.077
Na	0.073	**−0.112**
M_f_ FIV	0.069	**0.168**
K	0.068	0.081
Methyl dodecanoate	0.068	0.046
Ethyl dodecanoate	0.068	−0.027
M_n_ FIV	0.067	**0.167**
Isoamyl alcohol	0.061	−0.054
Geranyl ethyl ether	0.057	**0.125**
2,3-Butanediol	0.057	−0.019
Ferulic acid and derivatives	0.054	**−0.124**
Caffeic acid	0.052	**−0.166**
P	0.048	**0.124**
B	0.047	**0.134**
TOTAL ALCOHOLS	0.046	0.063
Free cinnamic acids	0.044	−0.018
Dodecanol	0.044	**0.107**
TOTAL TERPENES	0.042	0.019
Fertaric acid	0.040	**−0.146**
Diethyl succinate	0.038	0.051
Phenylethanol	0.033	**0.147**
Sinapic acid	0.031	−0.035
Sinapic acid and derivatives	0.031	−0.035
Ethyl coumarate	0.029	−0.062
Nerol oxide	0.027	0.021
Mg	0.027	**−0.157**
Tartaric Acid	0.026	−0.028
α Terpineol	0.017	0.032
Ferulic acid	0.010	**0.175**
b*	0.010	**−0.157**
C*	0.008	**−0.157**
Ethyl 2-methylbutyrate	0.008	0.055
*trans*-coumaric acid	0.000	**0.118**
L*	0.000	**0.191**
Color Intensity	−0.006	**−0.186**
Conductivity	−0.009	0.023
Conductivity Loss (%)	−0.010	**0.131**
Ethyl nonanoate	−0.014	0.077
FIII	−0.020	−0.027
Hotrienol	−0.025	**−0.129**
Linalol	−0.026	0.028
Citric Acid	−0.026	0.030
Zn	−0.032	**−0.131**
*cis*-coumaric acid	−0.043	−0.090
h*	−0.051	**0.131**
Coumaric acid and derivatives	−0.057	**0.139**
FIV	−0.063	**−0.155**
Titratable Acidity	−0.066	**0.150**
Ca	−0.067	**0.159**
Isobutanol	−0.067	**0.101**
Turbidity	−0.080	**−0.121**
FII	−0.091	0.012
Coutaric acid	**−0.100**	**0.141**
Caffeic acid and derivatives	**−0.100**	−0.053
Ethyl heptanoate	**−0.110**	−0.054
Malic Acid	**−0.114**	0.032
TOTAL cinnamic acids	**−0.114**	0.039
I_280_	**−0.129**	−0.047
Cu	**−0.130**	−0.007
Caftaric acid	**−0.132**	0.036
TOTAL polysaccharides	**−0.132**	−0.033
Tartaric esters of cinnamic acids	**−0.133**	0.064
FI	**−0.138**	0.051

Loadings greater than ± 0.100 are highlighted.

## Data Availability

No new data were created or analyzed in this study. Data sharing is not applicable to this article.

## Sample Availability

Samples of the compounds are not available from the authors.
